# 3D Porous Scaffold-Based High-Throughput Platform for Cancer Drug Screening

**DOI:** 10.3390/pharmaceutics15061691

**Published:** 2023-06-09

**Authors:** Yang Zhou, Gillian Pereira, Yuanzhang Tang, Matthew James, Miqin Zhang

**Affiliations:** 1 Department of Materials Science and Engineering, University of Washington, Seattle, WA 98195, USA; 2 Department of Neurological Surgery, University of Washington, Seattle, WA 98195, USA

**Keywords:** 3D scaffold, high throughput screening, rapid dispensing, pore size, drug resistance

## Abstract

Natural polymer-based porous scaffolds have been investigated to serve as three-dimensional (3D) tumor models for drug screening owing to their structural properties with better resemblance to human tumor microenvironments than two-dimensional (2D) cell cultures. In this study, a 3D chitosan–hyaluronic acid (CHA) composite porous scaffold with tunable pore size (60, 120 and 180 µm) was produced by freeze-drying and fabricated into a 96-array platform for high-throughput screening (HTS) of cancer therapeutics. We adopted a self-designed rapid dispensing system to handle the highly viscous CHA polymer mixture and achieved a fast and cost-effective large-batch production of the 3D HTS platform. In addition, the adjustable pore size of the scaffold can accommodate cancer cells from different sources to better mimic the in vivo malignancy. Three human glioblastoma multiforme (GBM) cell lines were tested on the scaffolds to reveal the influence of pore size on cell growth kinetics, tumor spheroid morphology, gene expression and dose-dependent drug response. Our results showed that the three GBM cell lines showed different trends of drug resistance on CHA scaffolds of varying pore size, which reflects the intertumoral heterogeneity across patients in clinical practice. Our results also demonstrated the necessity to have a tunable 3D porous scaffold for adapting the heterogeneous tumor to generate the optimal HTS outcomes. It was also found that CHA scaffolds can produce a uniform cellular response (CV < 0.15) and a wide drug screening window (Z′ > 0.5) on par with commercialized tissue culture plates, and therefore, can serve as a qualified HTS platform. This CHA scaffold-based HTS platform may provide an improved alternative to traditional 2D-cell-based HTS for future cancer study and novel drug discovery.

## 1. Introduction

Adult glioblastoma multiforme (GBM) is one of the most deadly and recalcitrant cancers in the U.S. [1]. For decades, extensive efforts have been dedicated to cancer drug development; yet, the failure rate and the cost of new drug discovery are exceptionally high [2,3]. One of the major obstacles in new drug development is that the conventional in vitro high-throughput screening (HTS) approach often fails to reliably predict biological responses in vivo [4]. HTS is a widely accepted practice in the pharmaceutical and biotech industry to quickly assess a large quantity of compounds in miniaturized in vitro assays [5]. Currently, the traditional two-dimensional (2D) in vitro HTS is the most common strategy for novel cancer therapeutics screening. Although relatively cheap and fast, the 2D cell culture cannot closely recapitulate the in vivo three-dimensional (3D) tumor tissue microenvironment, which regulates important cell–cell and cell–matrix interactions [3,6]. Consequently, the effective compounds selected by 2D screening often have a high failure rate in clinical trials and lead to slow and expensive drug development processes [3].

Compared to cancer cells cultured with 2D monolayers, cancer cells cultured in 3D matrix are able to form self-assembled aggregates, aptly termed “tumor spheroids”, and render cell behaviors similar to those in in vivo tumor tissues [7]. They have been shown to possess more physiological relevance to in vivo environments, and showed significantly increased cell malignancy, drug resistance and self-renewal abilities [4,8,9,10,11]. Therefore, much effort has been dedicated to developing 3D tumor spheroid models to improve the efficacy of the HTS of cancer drugs [4,12].

Natural polymers have been widely used in tissue engineering applications thanks to their excellent biocompatibility. Natural polymers, such as chitosan and hyaluronic acid (HA), contain similar chemical structures to glycosaminoglycan, a major extracellular matrix (ECM) constituent in the human body. These polymers are extensively studied and prove to be suitable for constructing 3D scaffolds for in vitro cell cultures as they provide a microenvironment that can closely mimic the human tissue ECM [13]. Compared to the commercialized, animal-sourced 3D matrices, such as Matrigel, these natural polymers provide a more economic option and pose less risk of incurring immunogenic responses [14,15,16]. In previous studies, 3D porous chitosan–hyaluronic acid (CHA) scaffolds have been utilized to successfully build 3D in vitro GBM tumor spheroid models [9,10,11]. Hyaluronic acid is abundant in human GBM tumor tissues and it has been demonstrated that it is associated with tumor growth and progression [17]. Yet, HA lacks proper mechanical strength that can support solid tumor growth in vitro. The negatively charged polymer backbone also hinders the initial cell attachment. Chitosan, another widely used cationic polymer for 3D matrix materials, can form polyelectrolyte complexes with HA and provide strong mechanical support in physiological conditions [13]. GBM cells cultured in CHA scaffolds exhibited elevated numbers of aggressive phenotypes, upregulated expression of cancer stem cell marker genes and enhanced drug resistance [9,11,13]. Thus, CHA scaffolds hold great promise as 3D HTS platforms for cancer drug development.

However, there are obstacles still holding the translation of such scaffold-based HTS platforms back, from bench-top research to human clinical trials. First, the polymer solution of chitosan and HA is usually highly viscous and difficult to handle, hampering the large-batch production of CHA scaffolds [18]. Hence, it is vital to establish a scalable manufacturing strategy that can accommodate the 3D CHA scaffold to a HTS platform. Second, human tumors exhibit significant heterogeneity in drug resistance, owing to their inter- and intra-patient genomic and phenotypic diversity [19]. Obtaining precise HTS results can be challenging when testing various types of cancer cells. Thus, it is essential for the scaffold to possess a customizable microenvironment capable of accommodating diverse cancer cell types, consequently optimizing the HTS outcome based on the characteristics of the tumor to be treated.

In this study, we applied a self-designed rapid dispensing system to manage the viscous polymer solution of chitosan and HA and establish a fast and cost-efficient approach to manufacture the HTS platform. We prepared the CHA scaffolds using the freeze-drying technique, which is suitable for processing heat-sensitive natural polymers and capable of controlling the porous structure of the scaffold by adjusting freeze-drying parameters [20]. Recent studies showed that changing the scaffold pore size alters the 3D microenvironment, affecting cell proliferation, migration, and differentiation [21,22,23]. We prepared CHA scaffolds of different pore sizes and explored whether the tunable CHA scaffold can regulate the drug resistance of different cancer cell lines. The growth kinetics, gene expressions and dose-dependent drug response of three GBM cell lines grown in the scaffold were assessed to reveal the effect of the scaffold pore size on the drug resistance. The coefficient of variance and Z′ factor were also analyzed to verify the quality of the scaffold as an HTS platform. We aim to develop a novel 3D scaffold-based HTS platform with great tunability to improve drug development efficiency and facilitate future cancer treatment.

## 2. Method

All chemicals were purchased from Merck (Rahway, NJ, USA), unless otherwise specified.

### 2.1. Scaffold Fabrication

A total of 8% of CHA scaffolds were prepared similarly to a previously described method [9]. A total of 8% *w*/*w* chitosan (Medical grade, Matexcel, Shirley, NY, USA) and 1% *w*/*w* hyaluronic acid (hyaluronic acid sodium salt, from *Streptococcus equi*) were fully dissolved in 1% *w*/*w* acetic acid aqueous solution, respectively. Two solutions were then combined and mixed using a Thinky mixer (ARM-300, Thinky, Laguna Hills, CA, USA) at 2000 rpm for three minutes, and further mixed in a blender for ten minutes to ensure a homogeneous polymer mixture. The polymer mixture was then centrifuged at 2000 rpm for 30 min to remove air bubbles and cast into 96-well tissue culture plates using a self-designed computer-aided automated scaffold dispenser [18]. The dispensing pressure and dispensing time were controlled to achieve a dispensing of 65 μL/well in order to reach a thickness of around 2 mm. The total dispensing time for each 96-well tissue culture plate is 40–50 s. To produce scaffolds with large pore sizes, the plates were first frozen at −20 °C for 24 h, then thawed at an ambient temperature for 2 h. The plates were then carefully transferred into a SP VirTis Genesis Pilot Lyophilizer (SP Scientific, Warminster, PA, USA). The plates were frozen in the lyophilizer using the following settings: 0 °C for 60 min, ramp to −5 °C in 40 min and stay for 20 min, ramp to −20 °C in 15 min and stay for 30 min and ramp to −2 °C in 18 min and stay for 24 h. To produce scaffolds with medium pore size, the plates were directly transferred into the lyophilizer and the following freezing setting was used: 0 °C for 60 min, ramp to −5 °C in 40 min and stay for 20 min, ramp to −20 °C in 15 min and stay for 30 min and ramp to −2 °C in 18 min and stay for 4 h. To produce scaffolds with small pore sizes, the plates were directly transferred into the lyophilizer and the following freezing setting was used: 0 °C for 60 min, ramp to −5 °C in 40 min and stay for 20 min, ramp to −70 °C in 65 min and stay for 30 min and ramp to −2 °C in 68 min and stay for 1 h. After freezing, the plates were lyophilized under 100 mTorr at −1 °C until the scaffolds were fully dehydrated. The scaffolds were neutralized in a 7% *v*/*v* ammonium hydroxide/methanol solution for 30 min under vacuum, rinsed intensively with DI water and soaked in PBS for 24 h to remove residual base. The scaffolds were then sterilized using 70% ethanol for 24 h and then washed with sterilized PBS three times and incubated in 37 °C for another 24 h prior to cell seeding.

### 2.2. Scaffold Characterization

Scaffold imaging and pore size characterization: Scaffolds were sectioned into 400 µm thin slices using a Compresstome VF-300 Vibrating Micromtome (Precisionary, Natick, MA, USA). Samples for SEM were sputter-coated with gold/platinum before imaging. SEM images were captured under 100× and 300× magnification using a JSM-6010 Plus scanning electron microscope (JEOL, Tokyo, Japan). Samples for optical imaging were hydrated with PBS for 7 days before imaging. Optical images were captured using a MU-1000 optical microscope (Amscope, Center Valley, PA, USA). The scaffold pore size was characterized by measuring the individual pore diameter using the ImageJ software program. At least 50 pores were measured for each scaffold.

Scaffold open porosity: The scaffold open porosity was measured using the liquid displacement method, as previously described [9,24,25]. Briefly, dry scaffold volume (*V_i_*) and weight (*W_i_*) were first recorded. The scaffold was then immersed in isopropanol (with known density *ρ_i_*) under vacuum until the scaffold stopped bubbling and sank to the bottom. The impregnated scaffold was carefully wiped off of excessive isopropanol and weighed again to obtain the final weight (*W_f_*). The change in the volume of the impregnated scaffold was deemed to be negligible as isopropanol is a nonsolvent. The open porosity was defined as the ratio of the volume of the solvent within the scaffold pores to the volume of the dry scaffold, as shown in Equation (1).

(1) $$Open\;Porosity=\frac{\left(W_{f}-W_{i}\right)/\rho_{i}}{V_{i}}\times 100\%$$

Mechanical testing: The scaffolds for compressive strength and modulus measurement were prepared in 24-well plates with the freezing setting described above. Scaffolds were hydrated with PBS and trimmed into cylindrical shapes of 10 mm in height and 14 mm in diameter. The compression test was conducted at room temperature using a Shimadzu universal tester (AGS-X Series, Shimadzu, Kyoto, Japan) with a rate of 0.4 mm/min until at least 50% strain was obtained. The compressive strength was determined as the compressive stress at the yield point. The compressive modulus was determined as the slope of the linear region of the stress–strain curve. The scaffolds for Young’s surface modulus measurement were sectioned into 400 µm thin slices and hydrated with PBS prior to measurement. Young´s surface moduli of scaffolds were obtained by conducting the nanomechanical measurement using a EasyScan atomic force microscope (Nanosurf AG, Liestal, Switzerland). The measurement was performed under an aqueous environment, using a ContAl-G (BudgetSensor, Sofia, Bulgaria) silicon nitride tip in a contacting mode. Each sample was scanned in an 8 × 8 array at three different 1 nm^2^ square areas. Young’s surface modulus was calculated based on force-displacement curves.

Zeta potential measurement: The zeta potential of scaffolds was analyzed using a SurPASS 3 electrokinetic analyzer (Anton Paar, Ashland, VA, USA). Briefly, scaffolds were cast and freeze-dried into 1 mm thick sheets in 60 mm Petri dishes. The scaffolds were then neutralized, rinsed and trimmed to fit the size of the sample tube of the electrokinetic analyzer. The zeta potentials of the scaffolds were measured in triplicate using 0.01 M KCl as a buffer solution at pH = 7.4 to reflect the surface charge in physiological conditions.

### 2.3. Cell Proliferation Analysis

The human glioblastoma cell lines U-87 MG and U-118 MG were purchased from American Type Culture Collection (ATCC, Manassas, VA, USA). The human glioblastoma cell GBM6 was previously established in our laboratory [11]. Cells were seeded on 2D 96-well plates and PBS-damped CHA scaffolds with different pore sizes in a 96-well plate at 5000 cells per well and cultured in a fully supplemented 100 µL medium (Dulbecco’s modified eagle medium with 10% fetal bovine serum and 1% antibiotic-antimycotic). The cell metabolic activities were monitored on Days 1, 2, 4, 7 after seeding using the AlamarBlue metabolic assay and following the manufacturer’s protocol (Life Technologies, Carlsbad, CA, USA) [26,27,28]. Briefly, the AlamarBlue stocking solution was diluted ten times using a fully supplemented medium and then added to each well (150 µL) and incubated at 37 °C for 2 h. Next, 100 µL of the AlamarBlue solution were transferred from the cell-culture plate to an opaque 96-well plate, and the fluorescence intensity was measured using a VersaMax Microplate Reader (Molecular Devices, Taunton, MA, USA). The cell number was calculated based on previously generated standard curves.

### 2.4. Cell Morphology Analysis

The cell morphology in scaffolds with different pore sizes was studied by observing the shape of tumor spheroid using live/dead imaging. The scaffolds were sectioned into 400 µm slices for better transparency prior to cell seeding. Cells were seeded on CHA scaffolds in a 96-well plate at 5000 cells per well, and cultured for 7 days in a fully supplemented medium. After Day 7, the fully supplemented medium was replaced by fluorescence dyes (PBS containing 0.1% *v*/*v* Calcien AM and 0.1% *v*/*v* PI), and then incubated for 30 min before imaging. Next, the scaffolds were mounted to microscope slides. A total of 10 μL of fluorescence dyes were added to prevent scaffolds from drying and covered with the coverslips immediately. Fluorescence images were captured using a Nikon TE300 (Nikon, Kyoto, Japan) inverted microscope.

### 2.5. PCR Analysis

Cells were seeded on 2D 96-well plates and scaffolds in 96-well plates at 5000 cells per well and cultured in a fully supplemented medium for 7 days. After Day 7, the cells were collected from 2D plates and scaffolds using TriplE express. The RNA was extracted using Qiagen RNeasy kit (Qiagen, Hilden, Germany), following the manufacturer’s protocols. The reverse transcription was conducted using the iScript cDNA synthesis kit (Bio-Rad, Hercules, CA, USA), following the manufacturer’s protocols to produce cDNA. Thermocycling was performed in a 20 μL solution system with 10 μL of SYBR Superrmix (Bio-Rad, USA), 2 μL of 10 nM primers, 7 μL of DNase-free water and 1 μL of 50 ng/μL cDNA. The qRT-PCR was conducted on a CFX96 Touch Real-Time PCR Detection System (Bio-Rad, USA). The thermocycle was set at 95 °C for 2 min, 40 cycles at 95 °C for 15 s, 58 °C for 30 s, and 72 °C for 30 s. Data were analyzed with the CFX Manager software (Bio-Rad, USA) with expression levels normalized to GAPDH. The primers (Integrated DNA Technologies, Coralville, IA, USA) are listed in Table 1.

### 2.6. TMZ Dose–Response Study

Cells were seeded on 2D 96-well plates and CHA scaffolds in 96-well plates at 5000 cells per well and cultured in a fully supplemented medium for 7 days. After Day 7, the fully supplemented medium was replaced by a TMZ solution with a gradient concentration of 0 (with 0.1% *v*/*v* DMSO), 10, 50, 100, 200, 500, 1000, 2000 μM and treated for 72 h. After 72 h, the cell metabolic activities were measured by the AlamarBlue assay, as previously described. The relative cell viability was determined as the relative metabolic activities of the untreated control groups.

### 2.7. Z′ Factor and CV Calculation

The screening window coefficient, Z′ factor, was calculated to evaluate the quality of the CHA scaffolds as an HTS platform. The coefficient of variance, CV, was also calculated to analyze the result consistency. The CHA scaffolds with medium pore sizes were used for HTS validation. Briefly, cells were seeded on 2D 96-well plates and 8% CHA scaffolds in 96-well plates at 5000 cells per well and cultured in a fully supplemented medium for 7 days. After Day 7, cells were treated with 2000 μM TMZ solution as the positive control, and 0 μM TMZ (with 0.1% *v*/*v* DMSO) as the negative control for 72 h. After 72 h, the cell metabolic activities were measured by the AlamarBlue assay, as previously described. The cell metabolic activities quantified by fluorescence intensities were used to calculate the Z′ factor and CV. The CV and Z′ for each cell line were obtained from three independent micro-plates. The CV was calculated by dividing the standard deviation of fluorescence intensity with average intensity. The *Z*′ was calculated using Equation (2).

(2) $$Z^{\prime }=1-\frac{3\left(\sigma_{p}+\sigma_{n}\right)}{\left|\mu_{p}-\mu_{n}\right|}$$

where *σ_p_* and *μ_p_* are defined as the standard deviation and mean of the fluorescence intensity in positive controls. *σ_n_* and *μ_n_* are defined as standard deviation and mean of the fluorescence intensity in negative controls.

### 2.8. Statistical Analysis

All data were presented as the mean ± standard deviation (SD) of the mean. *p* < 0.05 was set as statistical significance and was tested with Student’s *t*-test (GraphPad Prism, Boston, MA, USA).

## 3. Results and Discussion

### 3.1. CHA Scaffold Characterizations

#### 3.1.1. Fabrication of 3D Porous CHA Scaffolds as HTS Platform

**Figure 1:**
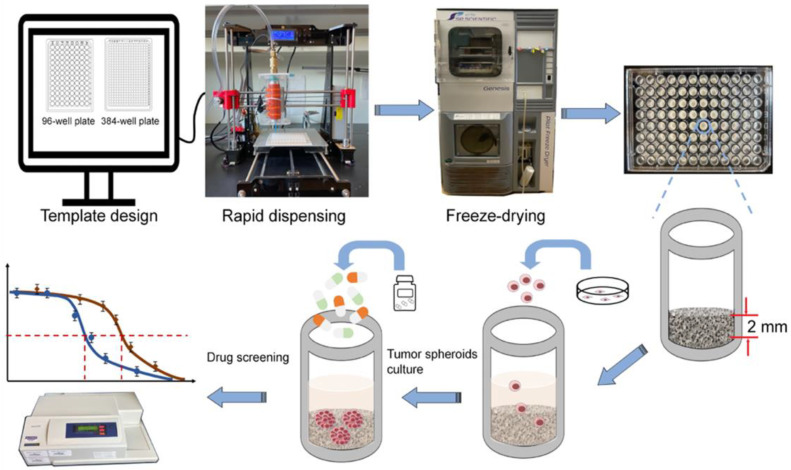
Schematic illustration of 3D CHA scaffold HTS platform manufacture and its application in drug screening.

**Figure 2:**
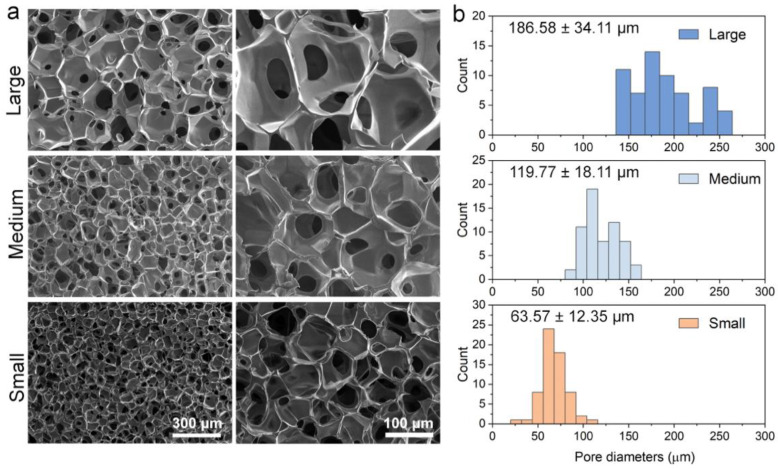
Structure characterization of CHA scaffolds of three different pore sizes. (**a**) From top to bottom, SEM images of scaffolds with large pore (CHA-L), medium pore (CHA-M) and small pore (CHA-S) under 100× (right column) and 300× (left column) magnification. (**b**) From top to bottom, the pore size distributions of CHA-L, CHA-M and CHA-S scaffolds. At least 50 pores were measured for each scaffold.

The HTS platform was manufactured by computer-aided rapid dispensing system of the CHA polymer solution and followed by freeze-drying to generate a 3D porous structure, as shown in Figure 1. The design and setup of the dispenser was presented in a previous work of our lab, where a fully automated and well-controlled dispensing system of viscous polymer solution into 96- and 384-well plates could be achieved [18]. As shown in Figure S1, the CHA scaffolds were uniformly cast as 2 mm discs at the bottom of 96-well plates. The rapid solution dispensing process takes less than one minute per plate and can be readily scaled up. The cost for both chitosan and HA is much lower than the cost of the materials for commercialized 3D cell culture matrices, such as Matrigel^®^ or Geltrex^TM^. Therefore, it holds great promise for economic 3D HTS platform manufacture. The different scaffold pore sizes were achieved by altering the freezing-annealing time (Figure S2), while keeping the same polymer concentration. Different freezing histories and annealing times can create distinct ice crystal structures in a chitosan–HA mixture, leading to a varied scaffold pore size after dehydration [29]. We first measured the density of all three scaffolds, and they showed a similar density of approximately 0.075 g/cm^3^ (Figure S3). We then examined the scaffolds with a scanning electron microscope (SEM) to reveal their different microscopic structures. As seen in Figure 2a, three CHA scaffolds showed very distinct pore sizes, while all comprised highly interconnected pores and uniform pore shapes. The scaffolds with large pore sizes (CHA-L), medium pore sizes (CHA-M) and small pore sizes (CHA-S) have an average pore diameter of ~186.58 µm, ~119.77 µm and 63.57 µm, respectively (Figure 2b). These results demonstrated the excellent tunability of the CHA scaffold structure created as a result of the freeze-drying method.

#### 3.1.2. Physical Properties of CHA Scaffolds

**Figure 3:**
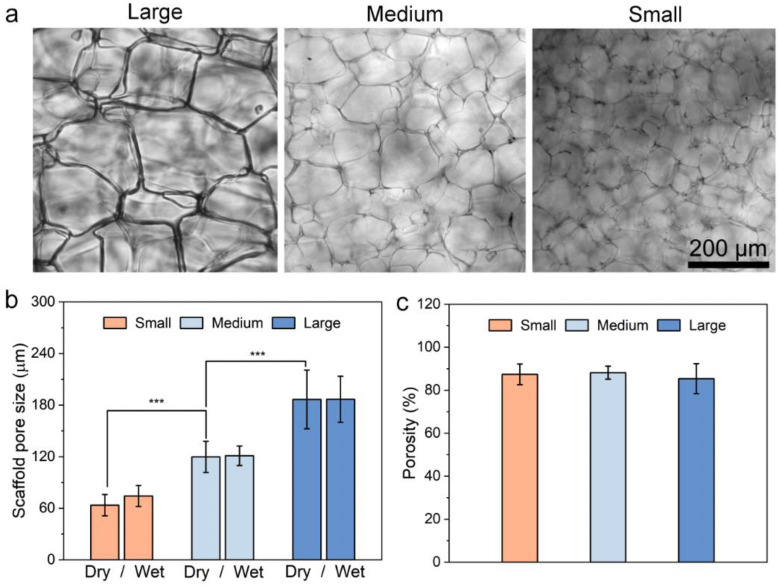
Porous structures of CHA scaffolds of three pore sizes. (**a**) Optical images of CHA scaffolds with large, medium and small pores after being hydrated by PBS for 7 days. (**b**) Scaffold pore size in dry and wet conditions. *n* > 10. (**c**) Porosity of scaffolds of three different pore sizes, measured by liquid displacement using isopropanol, *n* = 5. *** *p* < 0.001.

The structure stability of 3D porous scaffolds during cell culture is important for appropriate cell attachment, proliferation, and consistent cellular responses. It is imperative for scaffolds to maintain an intact pore structure, stable pore size and good interconnectivity under physiological conditions. Here, the structure stability of the scaffolds was first examined by comparing the hydrated scaffolds with the dry scaffolds. The CHA scaffolds were hydrated and incubated in PBS at 37 °C for 7 days before being imaged optically (Figure 3a). As shown, the CHA scaffolds of three different sizes (small, medium and large) incubated under physiological conditions remained uniform in pore structure one week after incubation in PBS. The hydrated scaffolds showed virtually no changes in pore size, as compared to their dry counterparts (Figure 3b), suggesting that the scaffolds bear excellent structure stability. Figure 3c shows a comparison of scaffold porosity between the scaffolds of three pore sizes. All three scaffolds showed high open porosity (~90%), and no significant difference in porosity was found. The high scaffold open porosity is essential to accommodate cell proliferation [22]. An open and interconnected pore inside the CHA scaffolds allows for the proper exchange and diffusion of nutrients, oxygen, and drug molecules, which support a healthy cell status and also an accurate cellular response to drug treatment.

**Figure 4:**
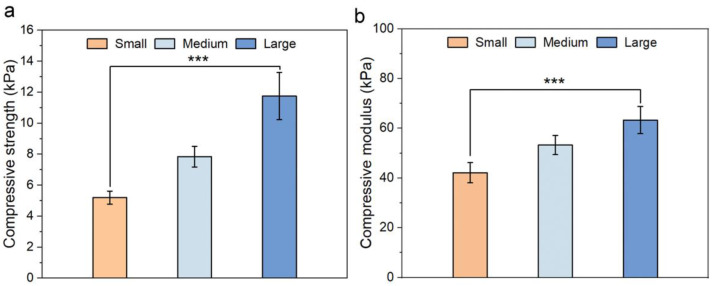
Mechanical properties of scaffolds characterized by compression test. (**a**) The average compressive strength of scaffolds of three different pore sizes (small, medium, large). (**b**) The average compressive moduli of scaffolds of three different pore sizes. *n* = 5. *** *p* < 0.001.

In addition to the stability, the stiffness of scaffolds has a strong influence on cell adhesion, morphology, as well as cell signaling pathways [30,31]. The mechanical environments sensed by cancer cells largely influence the cancer cell spread and metastasis [32]. Therefore, the mechanical properties of CHA scaffolds with different pore sizes were further evaluated. We first measured the compressive strengths and moduli of different CHA scaffolds. As shown in Figure 4a,b, the CHA scaffolds with the largest pore sizes have the highest compressive strength (11.74 ± 1.52 kPa) and modulus (63.30 ± 5.44 kPa), while the scaffolds with the smallest pore size have the lowest strength (5.19 ± 0.42 kPa) and modulus (42.17 ± 4.04 kPa). This agrees with the results of previous studies in that an increase in pore size is accompanied by an increase in stiffness for bulk scaffolds [23,33]. Nevertheless, Young’s surface modulus has been reported to actually determine how a single cell senses its surrounding matrix [34,35]. It was also reported that the local surface mechanical properties of the pore walls of scaffolds are independent of varying pore size [33]. Therefore, we further evaluated Young’s surface modulus of the scaffolds using atomic force microscopy (AFM), and we found nearly identical Young’s surface moduli measured for three scaffolds (Figure S4). Thus, it was suggested that cancer cells incubated in CHA scaffolds with different pore sizes experience a similar micro-mechanical environment. Furthermore, the surface charge of scaffolds may also alter cell adhesion, migration and proliferation behaviors by regulating the surface protein absorption [36]. We measured the zeta potentials of the three scaffolds, and they showed no significant difference under physiological pH (Figure S5), owing to that all three scaffolds were prepared by the same materials and concentration. These results provided clear evidence that the micro-mechanical environment and surface properties of CHA scaffolds are independent of changes in pore size, suggesting which would be the only factor that may alter the tumor microenvironment and regulate the cellular behaviors in the 3D CHA scaffolds.

### 3.2. The Effect of Scaffold Pore Size on GBM Cell Proliferation and Morphology

**Figure 5:**
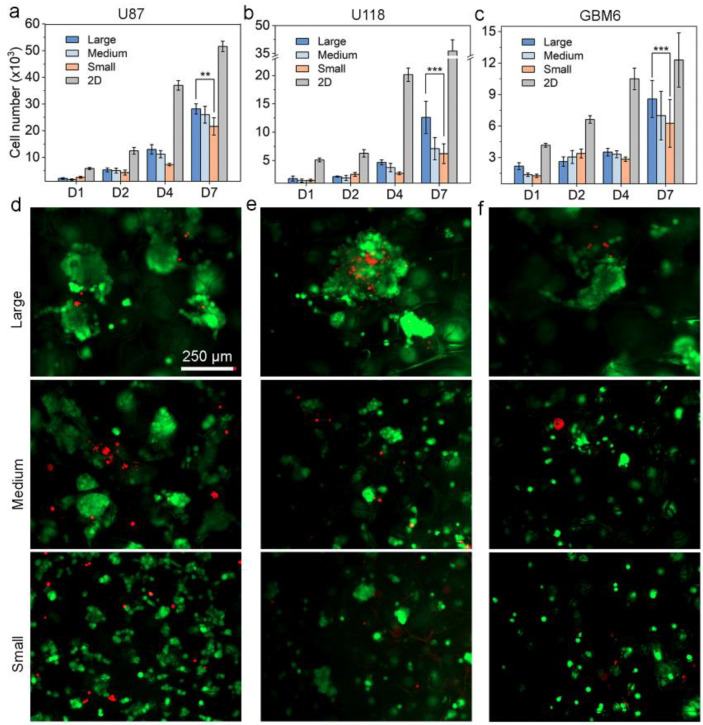
GBM cell proliferation profile and morphology on different substrates. (**a**–**c**) U87, U118 and GBM6 cell proliferation profiles on 2D micro-plates and in CHA scaffolds of small, medium, and large pore sizes, after 7 days of culture. *n* = 5. (**d**–**f**) Fluorescence images of live/dead cells of U87, U118 and GBM6 grown in CHA scaffolds with different pore sizes for 7 days. ** *p* < 0.01, *** *p* < 0.001.

Studies showed that the scaffold pore size can affect cell attachment [37], cell proliferation and migration [38]. We first investigated the influence of the pore size on the GBM cell proliferation. The GBM cell growth kinetics were monitored and compared among 2D (control) and 3D substrates with different pore sizes. Three human GBM cell lines, U87, U118 and GBM6, were chosen as they have been successfully utilized in in vitro tumor models generated in a 3D matrix for a tumor microenvironment study and a drug screening in recent studies [39,40,41]. Here, cells were seeded and cultured on both 2D plates and in 3D CHA scaffolds with different pore sizes for 7 days. The cell proliferation profiles are shown in Figure 5a–c for the 3 GBM cell lines, respectively. Three cell lines exhibited some similarities in that 3D cultures showed slower cell growth than 2D cultures. It was noted that scaffolds of large pore sizes (CHA-L) better supported GBM cell proliferation than scaffolds of medium and small pore sizes. Cell growth on different substrates after 7 days of culture were also visualized by fluorescence imaging with live/dead staining (Figure 5d–f). As seen, a substantial amount of tumor spheroids was formed in the CHA scaffolds of all pore sizes. Yet, the size of these tumor spheroids increased as the scaffold pore size increased. This is likely because the small pore size constrains the cell growth, as also demonstrated by other studies [42,43]. The diffusion limitation, greater surface area and smaller space in CHA-S might work collectively to hinder the cell attachment and proliferation [9,42,43]. It can be concluded that porous scaffolds with large pore size provide a beneficial microenvironment for cell proliferation.

### 3.3. The Effect of Scaffold Pore Size on Gene Expression of GBM Cells

**Figure 6:**
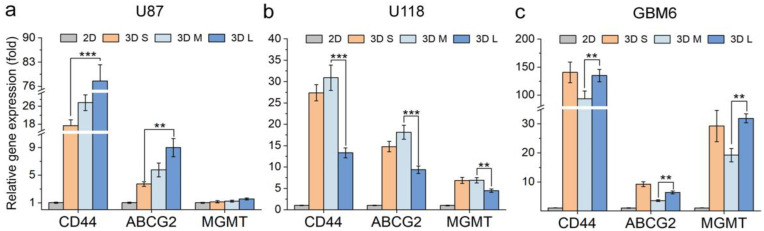
Gene expression of GBM cells cultured on different substrates for 7 days. The expression of drug-resistance-associated genes of (**a**) U87 cells, (**b**) U118 cells, and (**c**) GBM6 cells on 3D CHA scaffolds of different pore sizes relative to 2D culture, respectively. *n* = 3. ** *p* < 0.01, *** *p* < 0.001.

Besides cell proliferation, it is important to investigate the influence of the CHA scaffolds with varying pore sizes on the tumor drug resistance as a drug screening platform. Hence, the drug-resistance-associated gene expressions of the three cell lines cultured in the CHA scaffolds were further examined and compared with their 2D counterparts. Cells were cultured in CHA scaffolds for 7 days and then collected. Three tumor-malignancy-associated genes, the chemoresistance markers ABCG2 and MGMT and the tumor invasive marker CD44 were analyzed by RT-qPCR. The ABCG2 is an important member of the ABC-drug transporter family, which can actively efflux drugs from cells, serving to protect them from cytotoxic agents through ATP hydrolysis [44]. The MGMT encodes the DNA repair protein O6-alkylguanine (O6-AG) DNA alkyltransferase (AGT), which repairs the alkylating lesion caused by chemo drugs such as TMZ [45]. The CD44 represents an important cell surface receptor for hyaluronate, which modulates the tumor invasion and metastasis [46]. Figure 6 shows the expression of the above-mentioned genes for the three GBM cell lines cultured on different substrates relative to 2D controls.

Interestingly, the gene expression of the three GBM cell lines showed different trends in CHA scaffolds of varying pore sizes. For U87 (Figure 6a), the examined genes exhibited the highest expression in CHA-L scaffolds, followed by CHA-M in the middle and CHA-S scaffolds in the least, indicating that the larger pore size supports U87 cells to develop more drug-resistant and aggressive phenotypes. For U118 (Figure 6b), on the contrary, we observed the lowest gene expression in the CHA-L scaffolds, and the CHA-M had the highest expression level. For GBM6 (Figure 6c), the gene expression was highest in both CHA-L and CHA-S scaffolds, while the gene expression in CHA-M remained the lowest. The genetic heterogeneity of tumors has been long confounding clinical diagnoses and posing great challenges to the development of effective therapy for cancer treatment [47]. By changing the CHA scaffold pore size, we altered the tumor microenvironment, which is one of the major sources of tumor heterogeneity [47,48]. Comparing the three GBM cell lines grown in different CHA scaffolds, the gene expression was regulated differently in terms of the invasiveness, malignancy, and drug resistance genotypes, which presents a good example for intertumoral heterogeneity [47,48,49]. Therefore, by fine-tuning the scaffold pore size, we were able to better recapitulate the most malignant tumor in the in vivo environment and improve the reliability of the HTS outcomes in vitro.

### 3.4. Drug Response of GBM Cells Grown in Scaffolds of Different Pore Sizes

The cellular response of an in vitro tumor model to chemotherapy drugs is affected by a complex synergic effect of different tumor genotypes, hierarchies, cell–cell communications, and cell–matrix interactions [47,48,50]. The wide differences in the biophysical characteristics of different tumor cells present a significant challenge in determining the optimal HTS platform that can provide the best drug screening accuracy and efficacy [50]. As shown in the previous sections, the scaffold pore size is an important factor regulating an in vitro 3D tumor model, affecting the tumor proliferation and altering the gene expressions. Thus, a tunable scaffold pore size is essential in accommodating different cell lines for optimal drug screening outcomes.

Here, the drug resistance of the three GBM cell lines was further analyzed based on the cell viability under a dose-dependent drug response test. Three cell lines were cultured in three CHA scaffolds, and tested using a standard anti-cancer drug for GBM therapy, temozolomide (TMZ) [51,52]. The GBM cells were first allowed to grow in scaffolds for 7 days to adapt to different pore sizes and form tumor spheroids. The tumor spheroids were then exposed to TMZ for 72 h, and the dose-dependent cell viability was then evaluated as the percentage metabolic activity compared to non-treated controls on the same substrate. The half maximal effective concentration (EC_50_) was calculated to quantify the drug resistance.

**Figure 7:**
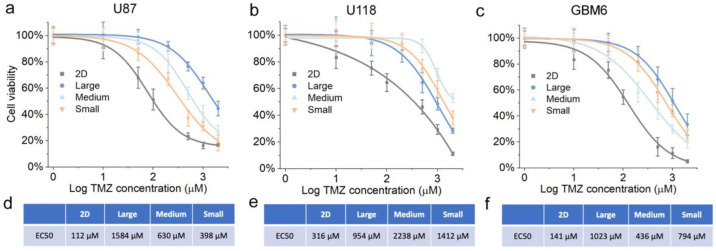
Drug response of GBM cells on different substrates. (**a**–**c**) Dose-dependent response of U87, U118 and GBM6 grown on different substrates after 7 days and treated with TMZ for 72 h. Cell viability was determined using AlamarBlue assay and normalized to untreated groups, *n* = 5. (**d**–**f**) EC_50_ for TMZ treatment of U87, U118 and GBM6 on 2D micro-plates and CHA scaffolds of different pore sizes.

Similar to the gene expression results, the three cell lines had distinct cellular responses to TMZ in scaffolds with different pore sizes. As shown in Figure 7a,d, the U87 showed the highest survival rate in the CHA-L scaffolds, followed by CHA-M and CHA-S. On the contrary, U118 showed the lowest survival in CHA-L, with CHA-M being in the highest and CHA-S being in the middle (Figure 7b,e). For GBM6, the cells in the CHA-L and CHA-S scaffolds had higher survival rates than those in the CHA-M scaffolds (Figure 7c,f). The three cell lines all showed higher survival rates in 3D CHA scaffolds than in their 2D counterparts regardless of pore size, providing evidence that the cells rapidly obtained different levels of drug resistance to TMZ in 3D cultures. Notably, the cell survival rates of the three GBM cell lines exhibited a good match with the drug-resistance-associated gene expression. These data further demonstrated that the drug resistance of an in vitro tumor model can be altered by tuning the pore size. Thus, the use of CHA scaffolds as an HTS platform allows us to create an in vitro tumor model with the highest level of malignancy, presenting unique opportunities to enhance drug screening efficacy.

### 3.5. Quality of 3D CHA Scaffold as an HTS Platform

**Figure 8:**
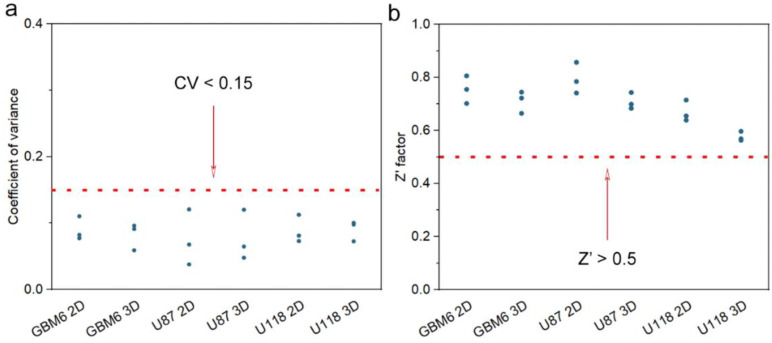
Characterization of the quality of CHA-scaffold-based HTS platform. (**a**) Coefficient of variance calculated for three GBM cell line viabilities in response to TMZ treatment on both 2D and CHA scaffolds with medium pore size, *n* = 3. (**b**) Z′ factor calculated for GBM cell viability in response to TMZ treatment on both 2D and CHA scaffolds with medium pore size, *n* = 3.

The criteria for assessing the quality of an HTS platform usually refers to two parameters: the cellular response uniformity and the drug screening window on 96- or 384-well-plate-based drug screening [53,54]. The uniformity is characterized by the coefficient of variance (CV), which is determined as the ratio of the standard deviation to the mean value of the cellular response across plates. The drug screening window is quantified by a coefficient termed “Z′ factor”, which reflects the data variation level and the cellular response signal dynamic range of control groups. The Z′ factor is an important characteristic parameter to assess the quality of an HTS platform based on properly selected positive and negative controls, without the intervention of test compounds [53]. Here, we investigated the quality of our CHA-scaffold-based HTS platform to compare with commercialized 2D tissue culture plates following a previously reported method with minor modification [54]. CHA scaffolds with medium pore size were picked as a sample platform; 0.1% DMSO and 2 mM TMZ were used as negative and positive controls for all three cell lines on both 3D and 2D cultures. As shown in Figure 8a, all three cell lines cultured in CHA scaffolds showed relatively low CV and were within the acceptable degree of variance for an in vitro cell-based assay (<0.15). In addition, the performance of the CHA scaffold platform was on par with commercialized 2D micro-plates. A Z′ factor above 0.5 was deemed as an excellent screening window between positive and negative controls [53]. As shown in Figure 8b, all three cell lines on 3D culture conditions showed a high Z′ factor value above 0.5. Together with the CV data, this proves that the CHA scaffold serves as a high-quality HTS platform comparable with commercialized 2D tissue culture plates.

## 4. Conclusions

In this study, we used a self-designed rapid dispensing system to achieve a fast and cost-effective large-batch production of 3D CHA porous scaffolds as a HTS platform. The 3D CHA porous scaffolds fabricated with adjustable pore size using a freeze-drying technique showed a significant difference in pore dimension, and a similar surface charge and micromechanical environment. We tested three GBM cell lines on the CHA scaffolds with different pore sizes and investigated the effect of pore size on the cell proliferation, morphology, and gene expression. Significantly, we performed a dose-dependent drug treatment using TMZ on the three cell lines in CHA scaffolds with varying pore size, and discovered that the results collectively capture the intertumoral heterogeneity of drug resistance. The heterogeneous nature of tumors is a major challenge in drug discovery and clinical trials. Our tunable HTS platform holds the potential of improving drug screening outcomes for different cancer cell lines by allowing us to adjust the pore size to achieve the highest drug resistance. In addition, we have demonstrated the high quality of the 3D HTS platform by validating comparable CV and Z′ factor values of three GBM cell lines tested with TMZ on CHA scaffolds and commercial tissue culture plates. Therefore, our 3D CHA-scaffold-based HTS platform stands as a powerful tool for future cancer research and new anti-cancer therapeutic development.

## Figures and Tables

**Table 1:** Primers used for PCR.

Target	Forward (5′–3′)	Reverse (5′–3′)
GAPDH	ACCACAGTCCATGCCATCAC	TCCACCACCCTGTTGCTGTA
CD44	CCAGAAGGAACAGTGGTTTGGC	ACTGTCCTCTGGGCTTGGTGTT
ABCG2	GTTCTCAGCAGCTCTTCGGCTT	TCCTCCAGACACACCACGGATA
MGMT	CCTGGCTGAATGCCTATTTCCAC	GCAGCTTCCATAACACCTGTCTG

## Data Availability

Data was generated during the study.

## Supplementary Materials

The following supporting information can be downloaded at: https://www.mdpi.com/article/10.3390/pharmaceutics15061691/s1, Figure S1. Optical photos of polymer solutions and freeze-dried scaffolds in 96-well tissue culture plates. Figure S2. Freezing profiles of scaffolds with different pore sizes. Figure S3. Density measurements of scaffolds of different pore sizes (Small, Medium, and Large). Figure S4. Scaffold surface Young’s modulus characterization by AFM. Figure S5. Zeta potential of scaffolds of different pore sizes measured at pH = 7.4.
