# Supplementary material for: 3D Porous Scaffold-Based High-Throughput Platform for Cancer Drug Screening
Source: Pharmaceutics. 2023 Jun 9;15(6):1691. doi: 10.3390/pharmaceutics15061691 (PMC10304563; doi:10.3390/pharmaceutics15061691)
Supplement: Supplementary file 1 [file pharmaceutics-15-01691-s001.zip › pharmaceutics-2400343-supplementary.pdf]

Supporting Information

3D porous scaffold-based high-throughput platform for cancer drug screening

Author name: Yang Zhou, Gillian Pereira, Yuanzhang Tang, Matthew James, and Miqin Zhang

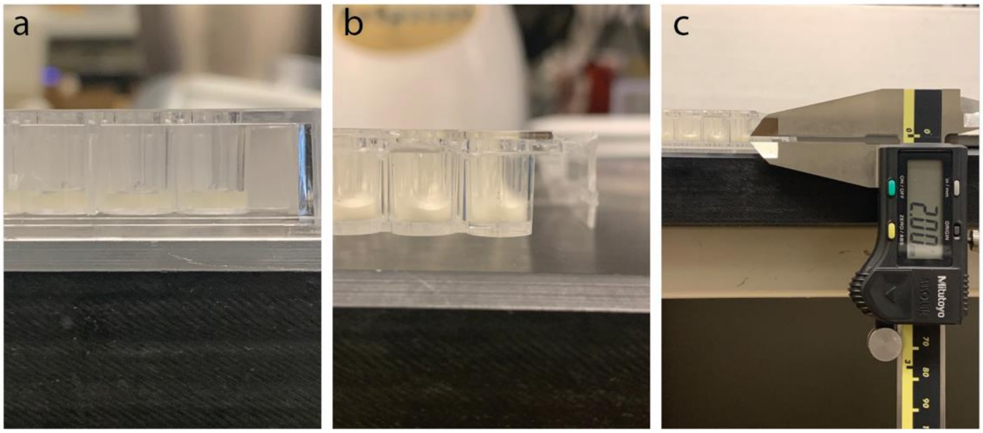

Figure S1. Optical photos of polymer solutions and freeze-dried scaffolds in 96-well tissue culture plates. (a) The picture of polymer solution dispensed in 96-well tissue culture plates. (b) The image of freeze-dried scaffolds in 96-well tissue culture plates. (c) The thickness measurement of the freeze-dried scaffolds using a caliper.

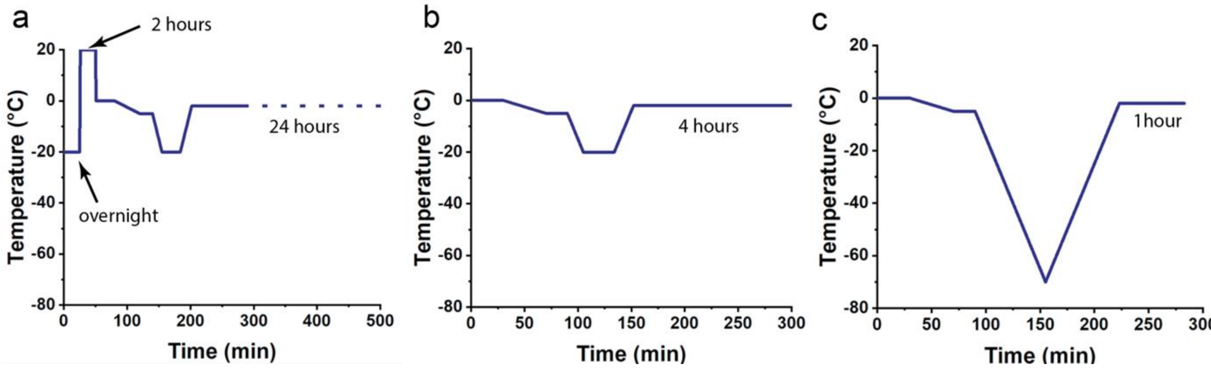

Figure S2. Freezing profiles of scaffolds with different pore sizes. (a) Freezing history of the scaffold with (a) large pore size, (b) with medium pore size and (c) smallest pore size.

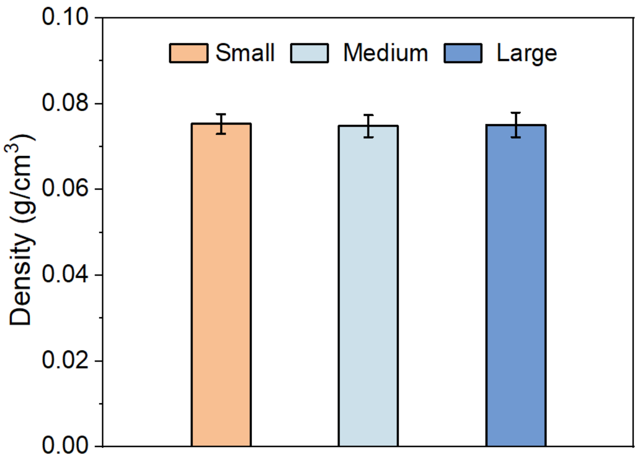

Figure S3. Density measurements of scaffolds of different pore sizes (Small, Medium, and Large). n = 5.

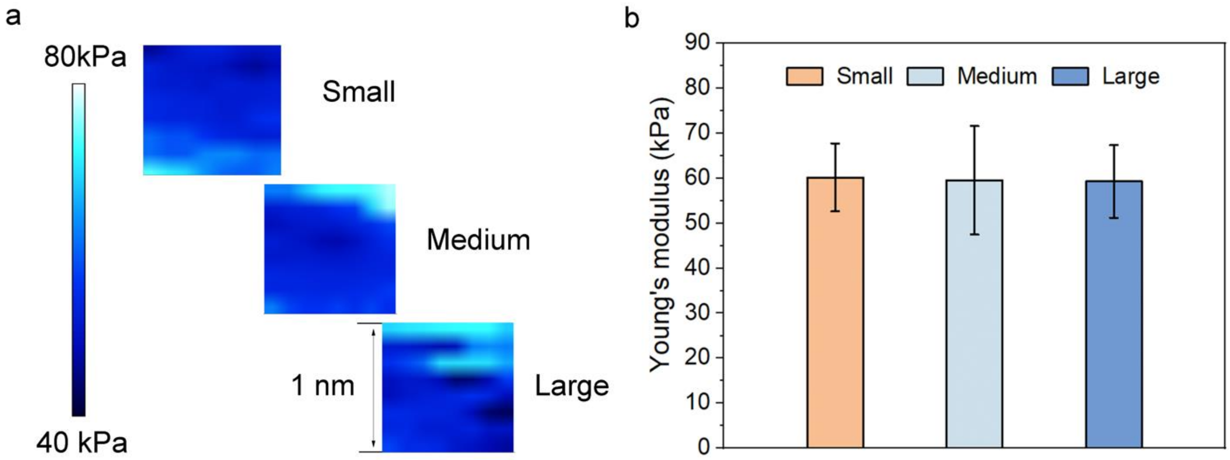

Figure S4. Scaffold surface Young's modulus characterization by AFM. (a) Surface Young's modulus mapping of 1×1 nm area on scaffolds of different pore sizes. (b) Average surface Young's moduli of scaffolds of different pore sizes. n = 3.

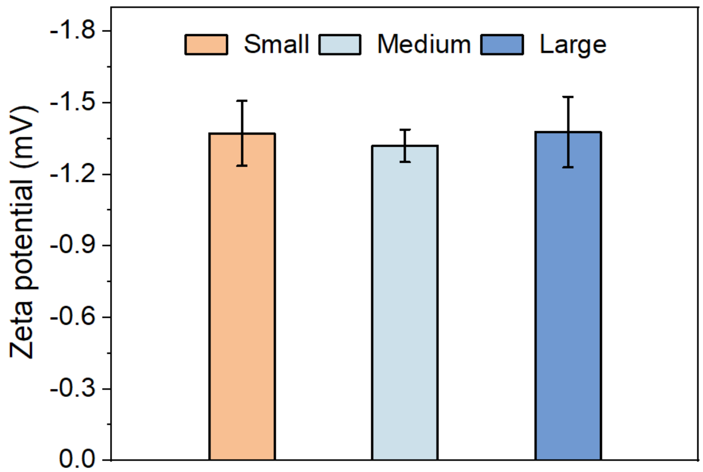

Figure S5. Zeta potential of scaffolds of different pore sizes measured at pH = 7.4. n = 3.
